# Treatment expectations and patient-reported outcomes of nusinersen therapy in adult spinal muscular atrophy

**DOI:** 10.1007/s00415-020-09847-8

**Published:** 2020-05-02

**Authors:** Alma Osmanovic, Gresa Ranxha, Mareike Kumpe, Lars Müschen, Camilla Binz, Flavia Wiehler, Lejla Paracka, Sonja Körner, Katja Kollewe, Susanne Petri, Olivia Schreiber-Katz

**Affiliations:** Department of Neurology, Hannover Medical School, Carl-Neuberg-Strasse 1, 30625 Hannover, Germany

**Keywords:** Spinal muscular atrophy (SMA), Patient-reported outcomes (PROs), Stanford expectations of treatment scale (SETS), Nusinersen, Antisense-oligonucleotide (ASO)

## Abstract

**Background:**

The antisense-oligonucleotide (ASO) nusinersen has recently been approved as the first genetically modifying therapy for 5q-associated spinal muscular atrophy (SMA) based on randomized sham-controlled trials in infants and children. The efficacy in adults with long disease history and advanced disease status is still widely unknown; the same applies to specific expectations of adult SMA patients and to what extent they are met and may impact outcome measures.

**Methods:**

In a longitudinal monocentric study in adult patients with SMA types 2–4, the Stanford Expectations of Treatment Scale (SETS) was assessed prior to and during nusinersen treatment. Treatment outcome was evaluated using patient-reported outcomes (PROs) as well as objectively quantifiable motor outcome measures.

**Results:**

Adult SMA patients had high expectations of nusinersen treatment effectiveness regarding increase in muscle strength and disease stabilization. Via PROs, 75% stated improvements in muscle strength, endurance and independence under therapy which was in line with slight improvements in quantifiable motor scores during a  ten month observation period. In contrast, patients only expressed few negative expectations which further decreased during therapy.

**Conclusions:**

This study showed mainly positive treatment expectations and PROs in patients undergoing nusinersen treatment along with measurable functional improvement in adult SMA patients. Moreover, treatment expectations did not significantly influence outcome measures.

**Electronic supplementary material:**

The online version of this article (10.1007/s00415-020-09847-8) contains supplementary material, which is available to authorized users.

## Introduction

Spinal muscular atrophy (SMA), a neuromuscular disorder with autosomal-recessive inheritance and the most common genetic cause of infant mortality, recently gained increased attention due to the approval of nusinersen, the first drug for treatment of SMA [1]. SMA is characterized by progressive degeneration of alpha motor neurons leading to progressive, disabling skeletal muscle weakness with scoliosis, respiratory insufficiency and reduced life expectancy. Patients are classified in different phenotypes depending on the age of symptom onset and the best motor milestone reached within the individual development (types 0–4) [2]. Herein, types 0 and 1 mark the most severe congenital/infantile SMA types, so far mostly lethal within the first years. SMA type 2 and 3 patients reach adulthood with a heterogeneous degree of motor function impairment. SMA type 2 patients manifest within the first 18 months of age. Approximately 70% of SMA 2 cases survive over 25 years, in the majority with a severe phenotype. SMA type 3 patients show a more variable phenotype. They acquire the ability to walk but may lose it during disease progression. Life expectancy is usually normal. The rare SMA 4 phenotype is characterized by adult-onset and overall milder condition [3, 4].

The main genetic cause of SMA (in > 95%) are homozygous deletions in the *SMN1* (survival of motor neuron) gene, resulting in a decreased expression of SMN protein, an essential protein for motor neuron maintenance [5]. An almost identical gene, *SMN2,* is present in multiple copies in the human genome. Due to impaired pre-mRNA splicing, *SMN2* produces only a small amount of intact SMN protein. A higher copy number of *SMN2* is related to a milder SMA phenotype and vice versa [5, 6]. Nusinersen, which specifically modifies *SMN2* splicing, has been approved for the treatment of all SMA subtypes based on two double-blind, sham-controlled, phase 3 studies conducted in infants and children up to nine years of age at the time of enrollment into the trial [7, 8]. Significant improvement of motor function by nusinersen treatment was demonstrated in both trials. Nusinersen, an antisense-oligonucleotide (ASO), does not cross the blood–brain barrier [9]. Therefore, the administration is performed by intrathecal injections  following a standard treatment regime, which encompasses initial loading doses on days 0, 14, 28 and 63 followed by maintenance therapy in 4-monthly intervals, as established in an open-label, phase 2 dose-escalating clinical study [10].

In adult SMA patients, controlled studies in larger patients groups assessing the efficacy of nusinersen with suitable outcome measures have not been conducted. A recently published observational study in a large cohort of nusinersen treated adults demonstrated significant improvements in the Hammersmith Functional Motor Scale Expanded (HFMSE) under nusinersen treatment [11]. Because of floor and ceiling effects in commonly used motor scores, and for monitoring of changes in motor-related symptoms such as ventilation and swallowing, patient-reported outcomes (PROs) have been discussed as an important additional outcome to assess treatment efficacy, especially in severely affected adult patients, in which common motor function tests are not applicable [11, 12]. However, patients’ individual outcome expectations of a specific treatment have been shown to influence treatment response, known as placebo effect [13, 14]. Sham-controlled efficacy trials in adult SMA patients are not feasible due to ethical reasons in respect of the unlimited approval of nusinersen. Patients’ expectations and self-reported outcomes under nusinersen treatment have not been studied yet. We aimed to evaluate adult patients’ expectations before and during nusinersen therapy, individually reported and quantitative outcome parameters and the relation between both in a prospective monocentric study.

## Methods

### Patients and clinical evaluation

We enrolled adult SMA patients aged 18 years and above treated with nusinersen at the Hannover Medical School between 2017 and 2019. The study has been approved by the local ethics review board. All patients gave their written informed consent before entering the study. A genetically confirmed diagnosis of SMA (homozygous deletion of exon 7 (or/and exon 8) of *SMN1*) was available for all patients prior to the initiation of treatment. Nusinersen was administered intrathecally (12 mg in 5 ml) in compliance with the recommended application scheme. Study enrollment took place either before or during treatment with nusinersen. Specific further baseline characteristics of all nusinersen treated patients were recorded, which included age, symptom onset, disease duration, *SMN2* copy number, SMA type, the ability to walk, presence of scoliosis, the need for non-invasive ventilation or a feeding tube and baseline muscle function impairment. The body mass index (BMI) was determined once at baseline. Muscle function was routinely assessed by professional therapists using the Revised Upper Limb Module (RULM) score [15] and the Hammersmith Functional Motor Scale Expanded (HFMSE) [16]. RULM has 20 items with a maximum of 37 points, higher scores indicating better upper limb function. The HFMSE is a validated 33 item scoring tool specifically for use in SMA patients. Each item is scored on a scale from 0 to 2, with a total of up to 66 points.

### Patient-reported decision-making process and treatment expectations

Patients were asked about their therapy decision-making process and they completed the Stanford expectations of treatment scale (SETS), a validated tool for measuring patients’ expectations regarding the outcome of a novel treatment, before and during nusinersen therapy. Each of the six items is coded with the same 7-point scale, starting from “I strongly disagree” up to “I strongly agree” [17]. Three items address positive (effectiveness, cure, confidence) and three items address negative (worries, fears, nervousness about negative effects) treatment expectations, respectively. Moreover, patients were asked to report their individual (positive and negative) expectations, which were summed up into reasonable categories afterwards. Longitudinal and cross-sectional analyses were performed.

### Patient-reported outcomes

Based on patient-reported expectations, we used a self-designed questionnaire to assess individual self-rated improvement and worsening during the therapy. Patients had to indicate improvement ordeterioration of 18 conditions compared to their medical condition before nusinersen treatment (supplementary material S1). An additional free-text option was provided. For analysis, each item was scored with one point, counted only once and visualized in a sum score as either improvement or worsening. Items in which significant treatment effects had been reported were identified afterwards and grouped into six main categories, named “muscle strength”, “endurance”, “independence”, “mobility”, “bulbar function”, “respiratory function”, further items were listed under “miscellaneous “. Patients also had the option to state that “nothing improved” or “nothing worsened”.

### Statistical analysis

Statistical analysis was performed using IBM SPSS Statistics 20® software. Differences between groups were analyzed by t-test or chi-square-test with a significance level of *p* < 0.05. Correlations were performed with Pearson or Spearman correlation. Univariate linear regression model analysis was performed if suitable.

## Results

### Patient characterization

24 adult SMA patients (nine females and 15 males) were enrolled into this study either at the start of (*n* = 16) or during nusinersen treatment (*n* = 8) with a mean age of 38.9 years and a mean disease duration of 31.1 years (Table 1). One SMA type 4 patient was further evaluated together with SMA type 3 patients. Ten patients were ambulatory, while another ten had a scoliosis and six needed a (part-time) non-invasive ventilation. The mean baseline motor scores were 23.2/66 for the HFMSE and 20/37 for the RULM score, both with a wide range which, altogether, reflects the various phenotypes and disease progression stages of the enrolled patients. Two SMA type 3 patients discontinued nusinersen treatment due to disease progression during the therapy, which was subjectively apparent and also confirmed by a decrease in motor scores. Side effects were reported in 96% (89% of SMA type 2 and 100% of type 3/4 patients) which were mainly related to the procedure of intrathecal administration (88%) like back pain and headache (Table 1).

**Table 1:** Characteristics of enrolled patients

*N* = 24	*N* (%)	Mean (SD)	Range
Women	9 (38)		
Age (y)		38.9 (13.5)	19.8–65.4
Age at therapy start (y)		37.9 (13.4)	19–64.4
Symptom onset (y)		6.8 (10.1)	0.5–47.2
Disease duration (y)		31.1 (14.2)	2.2–62.1
BMI		21.57 (6.1)	8.5–35.9
*SMN*2 copy number			
2	2 (8)		
3	10 (42)		
4	9 (38)		
5	1 (4)		
6	2 (8)		
SMA type			
Type 2	9 (38)		
Type 3	14 (58)		
Type 4	1 (4)		
Ambulatory	10 (42)		
Scoliosis	10 (42)		
Ventilation	6 (25)		
Feeding tube	2 (8)		
Motor function scores			
HFMSE (max. 66)		23.2 (25.1)	0–64
RULM (max. 37)		20 (12.8)	0–37
Treatment duration at analysis			
6 months	7 (29)		
10 months	9 (38)		
14 months	5 (21)		
18 months	3 (13)		
Patient enrollment			
At therapy start	16 (67)		
During therapy	8 (33)		
Side effects			
Administration-related			
Back pain	15 (63)		
Headache	14 (58)		
Nausea	4 (17)		
Vertigo	3 (13)		
Not Administration-related			
Constipation	2 (8)		
Upper airway infection	2 (8)		
Tachycardia	1 (4)		

*HFMSE* Hammersmith Functional Motor Scale Expanded,* max*. maximum, *N* number, *RULM* Revised Upper Limb Module, *SD* standard deviation, *SMN2* survival motor neuron 2 gene, *y* years

### Treatment information, decision-making and reasons for delay

We did not actively contact SMA patients to inform them on the approval of a new treatment. Our patients named the national patient registry (http://www.sma-register.de) [18] as the main information source (31% of enrolled patients) followed by information through friends (19%) and physicians (general practitioners, neurologists and others; 16%). Despite the awareness of nusinersen being the first drug to treat their condition, nearly 50% did not immediately opt for the treatment. The reasons were pending specialized medical/neurologist consultations (58%), own research (42%), private circumstances and fears of side effects (each 33%). In addition, uncertainty concerning study results and the administration procedure were stated (each by 25%) as reasons for decision delay.

Prior to treatment initiation, all patients presented at least once in our neuromuscular outpatient clinic. In this setting, they were informed about the results of the clinical trials ENDEAR and CHERISH with beneficial outcomes in children, adverse events (low platelet count, kidney disease, and hydrocephalus) and the administration procedure via lumbar puncture and its risks.

### Patient-reported treatment expectations

**Fig. 1:**
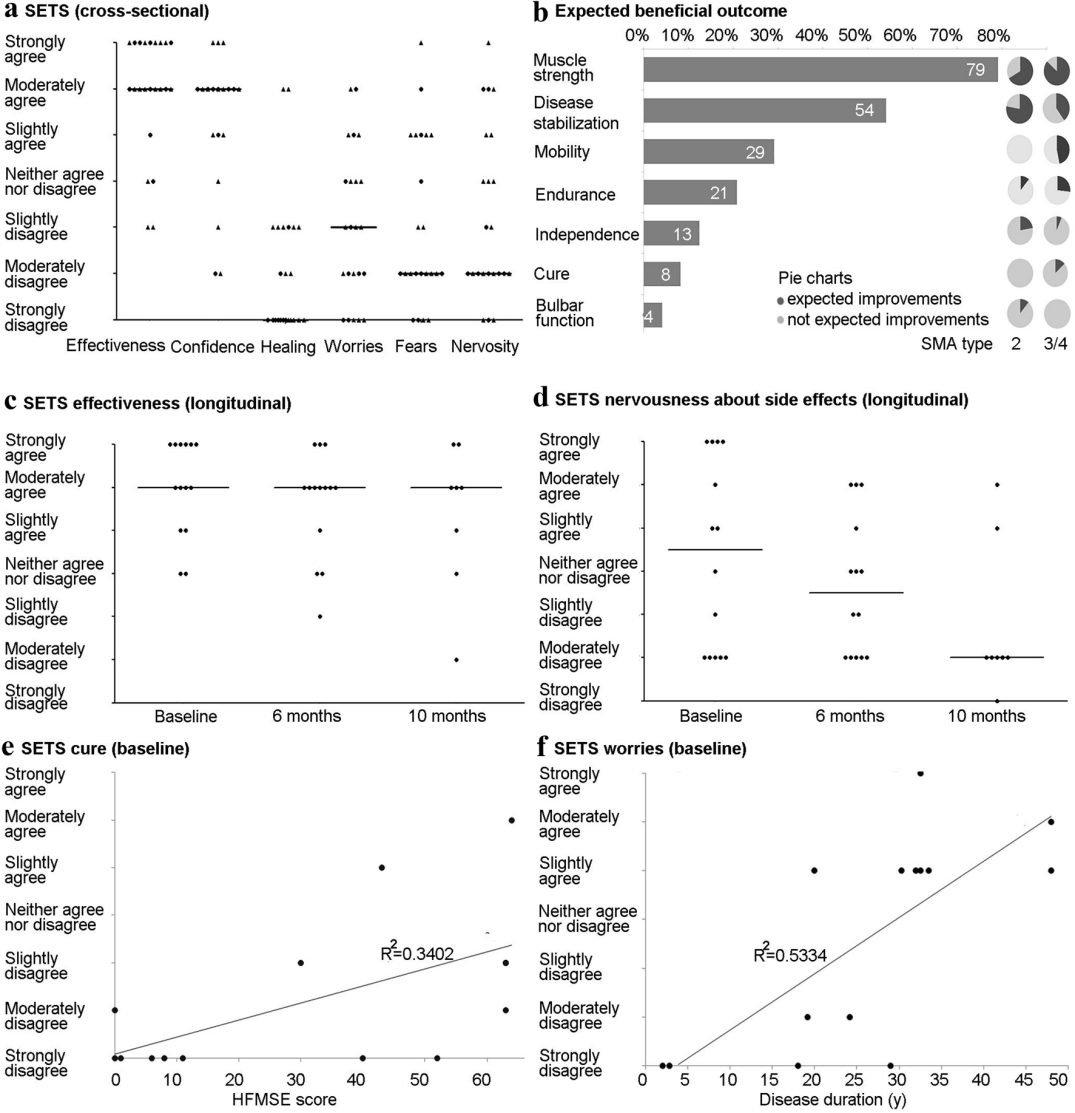
Treatment expectations of adult SMA patients. **a** Cross-sectional study of the six-item Stanford Expectations of Treatment Scale (SETS) within the first year of nusinersen treatment (*n* = 23; 6-months time point *n* = 19, ten months time point *n* = 4). Dots mark SMA type 3 or 4 patients, triangles mark SMA type 2 patients. The horizontal lines indicate all patients’ median for each domain. **b** Individually expected beneficial outcomes of nusinersen therapy (in % of *n* = 24 patients). Pie charts visualize the proportion of particular expected outcomes in either SMA type 2 (*n* = 9) or and type 3/4 (*n* = 15) patients. **c** Longitudinal analysis of the expectation of nusinersen effectiveness (baseline to month 10; *n* = 13). The horizontal lines indicate the median at each time point. **d** Longitudinal analysis of patients’ nervousness about side effects along with nusinersen therapy (baseline to month 10; *n* = 13). Again, the horizontal lines indicate the median at each time point. **e** The linear regression analysis displays the significant correlation of a higher HFMSE (Hammersmith Functional Motor Scale Expanded) score, thus a milder condition, with an increased expectation of a cure (*R*^2^ = 0.3402, *p* = 0.029). **f** The adjustment curve of the linear regression analysis pictures the significant relationship of patients’ increased worries about nusinersen treatment to a longer disease duration measured in years (y) (*R*^2^ = 0.5334, *p* = 0.003)

23 of 24 SMA patients (SMA type 2 *n* = 8, type 3/4 *n* = 15) completed the SETS questionnaire within the first year of treatment (Fig. 1a). Regarding positive expectations, 83% of patients expected effectiveness of nusinersen treatment and 83% stated to have complete confidence in this treatment. However, the majority of patients (91%) did not expect that the condition would be completely cured after the treatment. There were considerably less negative expectations: Only 22% of patients had worries, 30% expressed fears and 26% indicated to be nervous about negative effects of this treatment. No significant differences between SMA types were detected.

Specific individual expectations and therapeutic goals were obtained from all 24 SMA patients. 88% of the reported expectations could be summarized in seven categories (Fig. 1b). The majority of patients hoped for beneficial effects on muscle strength (79%) and disease stabilization (54%), followed by increased mobility, endurance and independence. An improvement in bulbar function was only expected by 4%, which only applied to SMA type 2 patients. Moreover, only 8% expected to be cured entirely (only SMA type 3 patients). Further expectations were only stated once or twice and therefore negligible (general improvements, facilitation in everyday life, improvement of mental health, support of SMA research and use of regular cutlery).

A subgroup of 14 SMA patients completed the SETS before therapy initiation. Pre-treatment expectation of nusinersen effectiveness was high and remained stable during the therapeutic course with no significant differences over time (Fig. 1c). However, major changes in negative expectations, such as nervousness about negative effects of nusinersen were seen during therapy. Pre-treatment nervousness notably decreased within the first year of nusinersen treatment (Fig. 1d).

Correlation analysis of pre-treatment expectation of effectiveness of nusinersen and age (*p* = 0.236), disease duration (*p* = 0.615), SMA type (*p* = 0.634), and disease severity (HFMSE *p* = 0.887, RULM *p* = 0.793) showed no significant relation. However, pre-treatment expectation of a cure of SMA by nusinersen revealed a significant correlation with baseline items of disease severity (HFMSE *p* = 0.029; RULM *p* = 0.035; *SMN2* copy number > 4 *p* = 0.030). Regression analysis confirmed the relationship between the HFMSE score and pre-treatment expectation of a complete cure of the disease (*R*^2^ = 0.3402) (Fig. 1e). Patients with a milder phenotype (thus higher *SMN2* copy numbers, higher  HFMSE and RULM scores) indicated greater expectation of a cure of their condition by nusinersen treatment. A significant correlation between disease duration and negative expectations (worries *p* = 0.003, fears *p* = 0.04 and nervousness about negative effects *p* = 0.021) was observed. Regression analysis indicated a linear relationship between disease duration and worries about the therapy (*R*^2^ = 0.5334) (Fig. 1f). Patients with longer disease duration indicated more negative expectations when asked before treatment initiation. This relationship disappeared within the first year of treatment, as negative treatment expectations considerably decreased during treatment.

### Patient-reported and motor outcomes

**Fig. 2:**
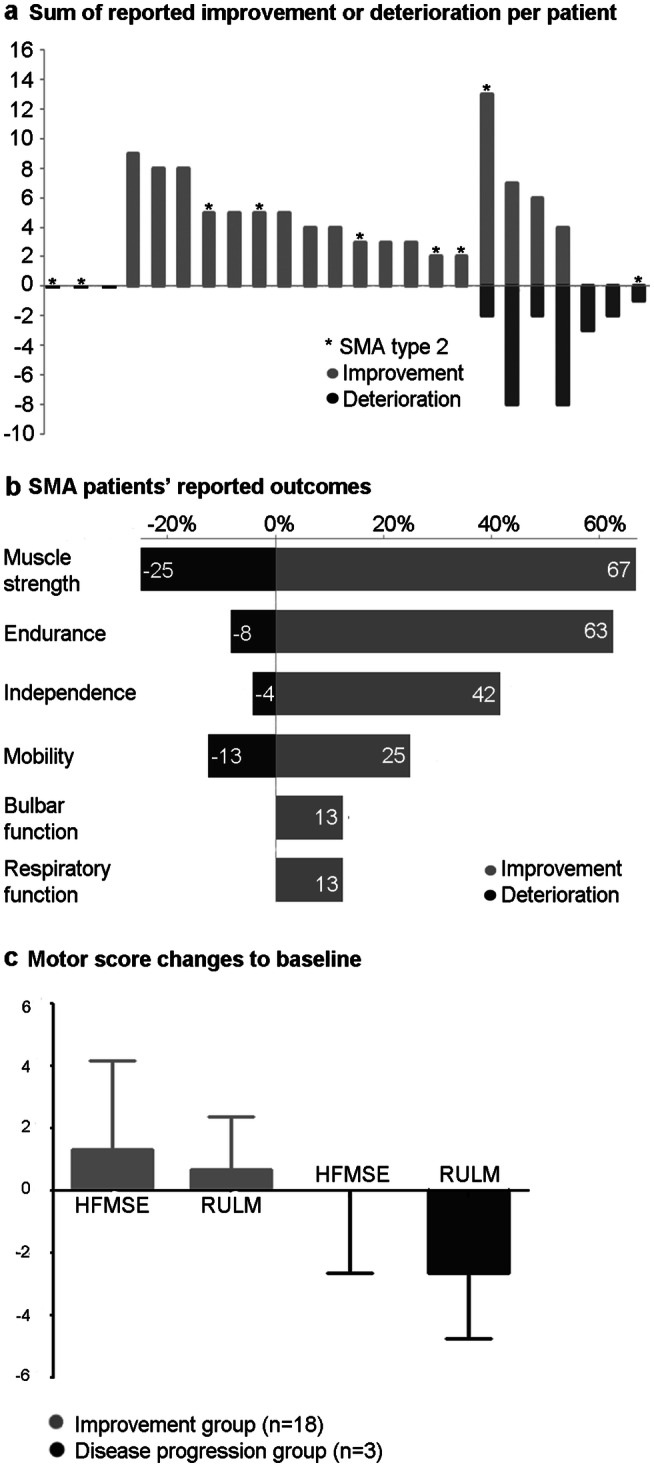
Patient-reported outcomes (PROs) and quantitatively measured motor scores under  ten months of nusinersen treatment. **a** Sum of reported improved and worsened conditions under nusinersen treatment (each bar indicates one patient). Light columns indicate improvements, dark columns deteriorations. * = SMA type 2 patients. **b** Ratio of patients who indicated improvement (in %) and deterioration (in %) of specific symptoms. Light bars represent improvement, the dark ones deterioration. **c** Changes in the quantitatively measurable motor scores  HFMSE (Hammersmith Functional Motor Scale Expanded) and RULM (Revised Upper Limb Module) under  ten months of treatment. Light bars represent patients who indicated subjective improvements (*n* = 18; HFMSE mean + 1.3, SD = 2.8; RULM mean + 0.7, SD = 1.7). Dark bars represent those patients who only stated worsening, which means disease progression (*n* = 3; HFMSE mean 0, SD = 2.6; RULM mean = − 2.7, SD = 2.08)

Three patients (13%) reported disease stabilization, which means neither improvement nor worsening, whereas 75% stated improvement during a follow-up of ten months under nusinersen treatment (Fig. 2a). An increase of muscle strength in either legs, arms, trunk or in general was more often reported in less severely affected patients (87% of SMA 3/4 vs. 33% of SMA 2) (Fig. 2b). In contrast, improvement of endurance, independence and mobility did not show specific differences according to SMA subtypes. 13% experienced an improved respiratory and bulbar function, both mainly in SMA type 2.

29% (seven cases) reported symptom worsening. In four out of these seven cases, both, deterioration of certain symptoms and improvement of others were reported. Three patients however stated only worsening, thus a disease progression can be assumed (Fig. 2a). Interestingly, 5/7 patients in this symptom worsening group had a SMA 3/4 subtype, who reported decrease in muscle strength, mobility, endurance and independence. Deterioration of bulbar or respiratory function was not reported under nusinersen treatment (Fig. 2b).

The mean objective motor scores, HFMSE [+ 1.0 (SD 2.6)] and RULM [+ 0.5 (SD 2.3), both increased during a treatment period of maximum 18 months throughout the entire patient cohort. Patients with subjective improvements (*n* = 18, see Fig. 2a), correspondingly developed a greater increase of motor scores, namely HFMSE + 1.3 (SD 2.8) and RULM + 0.7 (SD 1.7)] (Fig. 2c).

Only the BMI was significantly correlated with disease deterioration (*r* = 0.474, *p* = 0.019): regression analysis revealed that a higher BMI was significantly associated with more frequent reporting of worsening (*R*^2^ = 0.225). However, no significant correlation with objective motor outcomes (HFMSE and RULM) was detected. For age, SMA type, *SMN2* copy number, disease duration and ambulatory status or baseline motor scores no significant correlation was found (data not shown).

Moreover, we did not see any significant influence of pre-treatment expectations of nusinersen effectiveness on subjectively experienced outcome (improvement *p* = 1.00, worsening *p* = 0.076) or objective outcome measures (HFMSE *p* = 0.716, RULM *p* = 1.000). Treatment outcome therefore appeared to be not significantly biased by prior expectations.

## Discussion

Altogether, in this study we demonstrated that adult SMA patients had high expectations of treatment effectiveness and high confidence in nusinersen that remained stable during the treatment. Negative expectations (worries, fears, and nervousness about negative effects) were much less frequent and even further decreased during treatment. The most frequently reported expectations were an increase in muscle strength and a disease stabilization, whereas patients with a milder phenotype more often expected a cure of their condition. Analyzes of PROs support the efficacy of this therapy in adult 5q-associated SMA, as a higher proportion of patients (75%) reported an improvement of their disease status (increase in muscle strength, endurance and independence) compared to those who reported a symptom progression over an evaluation period of ten months. This was supported by an increase in the quantitative motor scores HFMSE (+ 1.3) and RULM (+ 0.7). Interestingly, pre-treatment expectations were not found to influence the outcomes under therapy in our cohort.

In 2017, when the first targeted treatment for SMA became available in Europe, placebo-controlled efficacy trials in adult SMA patients were missing. Medical information and outcome expectations were derived from clearly beneficial results in children (up to 12 years) with SMA type 2 and 3 who demonstrated clinically significant improvement in motor function in the HFMSE score over 15 months of treatment [8]. Consistently, in our cohort, 79% of adult SMA patients expected an increase in muscle strength. Nevertheless, the individual expectations were quite moderate and reflected the rather advanced disease stages decades after symptom onset.

Despite the necessity of intrathecal administration, markedly more positive than negative expectations were expressed by the patients and consistently, hardly a patient declined nusinersen treatment. Fear of negative effects and nervousness seemed to be of minor importance and even decreased within the course of the therapy. These data indicate a high acceptance and good tolerability of this therapy despite the burdensome intrathecal administration and high individual efforts (such as long distances to specialized centers, inpatient treatment, radiation exposure in case of computer tomography (CT)-guided administration and others). Interestingly, patients with a longer disease duration reported significantly more often negative pre-treatment expectations. In late-stage SMA, patients have severe thoracolumbar scoliosis [19], thus intrathecal nusinersen injection is substantially more challenging and patients might expect more side effects. In our daily routine and as described by others, CT-guided intrathecal delivery of nusinersen in SMA patients with severe scoliosis has been well established. This approach was shown to be safe and well tolerated [20–24]. However, side effects reported in our cohort and other recently published studies mainly resulted from the lumbar puncture procedure itself and were not directly related to the medication [24, 25]. The overall low occurrence of side effects and the high level of confidence in nusinersen therapy may underpin our observation of a constant decrease in negative treatment expectations during treatment.

So far, it is not well known to what extent patient expectations influence outcomes. However, pre-treatment expectation has been described as a factor that impacts treatment outcome. Individuals who strongly believe that they will benefit from treatment may be more likely to report benefits, known as placebo effect [13, 17]. Nusinersen has been approved for all SMA patients without limitations regarding the type of disease or patients’ age and disease duration; therefore, no placebo (untreated) control population is available in adults. As nusinersen is the first approved therapy for SMA, a placebo effect may be assumed. Our data, however, do not show a significant correlation between pre-treatment positive expectations and beneficial treatment outcome. To note, only 14 adult SMA patients completed the SETS before therapy initiation. Due to this small patient size at least a partially meaningful correlation cannot be fully excluded.

Regarding treatment efficacy, definite conclusions cannot be drawn from this study. We did, however, see indications for a beneficial effect. 75% of our patients reported clinical improvements such as an increase in muscle strength (67%), endurance (63%) and independence (42%). These improvements were not correlated to any baseline characteristics. Objective motor outcome scores, HFMSE and RULM, revealed the same trend. However, only a three point change in HFMSE is considered as clinically meaningful [16], which apparently stands in discrepancy to the patients’ subjectively reported improvement of muscle strength, possibly due to a certain placebo effect. Nonetheless, the natural course of SMA is known to be chronically progressive without spontaneous, even minor, improvements [26–29]. Moreover, the HFMSE does not reflect all relevant muscle functions. Changes in the 3-point scoring system mainly reflect gross motor function changes, as a score of 0 means “unable to perform the task” (i.e. item 1: “unable to sit”), a score of 1 means “perfoms the task with modification/adaptation/compensation” (i.e. item 1: “needs one hand support to maintain balance for a count of 3”) and score of 2 means “performs the task without modification/adaptation/compensation” (i.e. item 1: “able to sit using no hand support for a count of 3 or more”). Minor improvements, such as an increase in thumb movement, which may have a meaningful impact on a patient’s independence and communication/ability to work, are thus not captured by the HFMSE and also would be missed by the RULM score. Noteworthy, a 3-year follow-up study on nusinersen treatment in later-onset children with SMA and recently published studies conducted in adult SMA patients, observed significant improvements in motor function. In line with our data, these results provide evidence for the long-term benefits of nusinersen in later-onset SMA [11, 30, 31]. In our cohort, however, we also identified three patients who reported a deterioration of symptoms during the treatment (1 SMA type 2 and 2 SMA type 3) in line with a decline in quantitative motor function scores. Two of these patients have withdrawn from treatment so far.

Remarkably, our data demonstrate a significant correlation between BMI and patient-reported clinical worsening (*p* = 0.019). A higher BMI was significantly associated with more frequently reported worsening during nusinersen treatment. Treatment regimen and dosing for adults were adapted from the CHERISH and the ENDEAR trials in infants and children [7, 8, 10] but might not be sufficient for adult patients with higher body weight. A possible conclusion could be that weight-adjusted treatment regimens might have additional benefits. Treatment effects and body weight, however, did not correlate with HFMSE and RULM scores. Therefore, further studies addressing potential weight-dependent treatment response need to be performed.

Overall, the results of our study certainly must be confirmed over a longer time period and in larger patient cohorts. In rare diseases like SMA, this is only feasible within national and international collaborations [12, 32]. Another limitation is that the patient questionnaire used in this study to assess improvement or deterioration of symptoms has not been validated and can therefore not be used for comparisons between different studies. Further, it was not suitable to measure the quality of the reported outcomes but only indicated whether or not patients experienced improvement or worsening. Thus, standardized questionnaires for adult SMA patients are urgently needed. Our study demonstrates the advantages of PROs: small (subclinical) changes are detected more sensitively instead of being missed in assessments which only address gross motor changes. They might, in particular, be more sensitive in more severely handicapped patients in advanced disease stages. Moreover, PROs can be used as a quick and easy bedside test without special equipment or training.

To summarize, we systematically assessed the expectations and PROs of adult 5q-associated SMA patients under nusinersen treatment for the first time. We were able to demonstrate mainly positive expectations and a high level of confidence under therapy. Moreover, our data indicate the efficacy of nusinersen in adult, often severely affected patients with SMA types 2 and 3/4 and show that positive expectations were met in the vast majority of cases. Besides, we suggest PROs as an important measure to assess treatment effects. A multicenter approach and longer observation periods are needed for further confirmation.

## Electronic supplementary material

Below is the link to the electronic supplementary material.

Supplementary file1 (PDF 237 kb)
