# Supplementary material for: Treatment expectations and patient-reported outcomes of nusinersen therapy in adult spinal muscular atrophy
Source: J Neurol. 2020 May 2;267(8):2398–407. doi: 10.1007/s00415-020-09847-8 (PMC7359174; doi:10.1007/s00415-020-09847-8)
Supplement: Supplementary file 1 — Supplementary file1 (PDF 237 kb) [file 415_2020_9847_MOESM1_ESM.pdf]

Treatment expectations and patient-reported outcomes of nusinersen therapy in adult spinal muscular atrophy

Alma Osmanovic, MD[*1]; Gresa Ranxha[*1]; Mareike Kumpe[1]; Lars Müschen, MD[1]; Camilla Binz, MD[1]; Flavia Wiehler, MD[1]; Lejla Paracka, MD[1]; Sonja Körner, MD[1]; Katja Kollewe, MD[1]; Susanne Petri, MD[#1]; Olivia Schreiber-Katz, MD[#1]

[1] Department of Neurology, Hannover Medical School, Hannover, Germany

Correspondence to

Dr. Alma Osmanovic,
Department of Neurology
Hannover Medical School
Carl-Neuberg-Straße 1
Hannover, 30625, Germany
Phone +49 511 532 2392
Fax +49 511 532 3115
Email: Osmanovic.Alma@mh-hannover.de
ORCID: 0000-0002-6012-8423

S.1

Have you noticed any changes compared to your condition before starting with nusinersen therapy? YES/NO

If yes: Improvement ☐
Worsening ☐

Which improvements?: /*please mark with a cross*

General force	☐	Speech	☐
Muscle strength of the legs	☐	Respiration	☐
Muscle strength of the arms	☐	Locomotion	☐
Muscle strength of the trunk	☐	Independence in daily activities	☐
Endurance	☐	Falls	☐
Mobility	☐	Transfers	☐
Pain	☐	Independent dressing	☐
Cramps	☐	Independent hygiene	☐
Dysphagia	☐	Independent feeding	☐
		Nothing	☐

Other:

Which worsenings?: /*please mark with a cross*

General force	☐	Speech	☐
Muscle strength of the legs	☐	Respiration	☐
Muscle strength of the arms	☐	Locomotion	☐
Muscle strength of the trunk	☐	Independence in daily activities	☐
Endurance	☐	Falls	☐
Mobility	☐	Transfers	☐
Pain	☐	Independent dressing	☐
Cramps	☐	Independent hygiene	☐
Dysphagia	☐	Independent feeding	☐
		Nothing	☐

Other:
